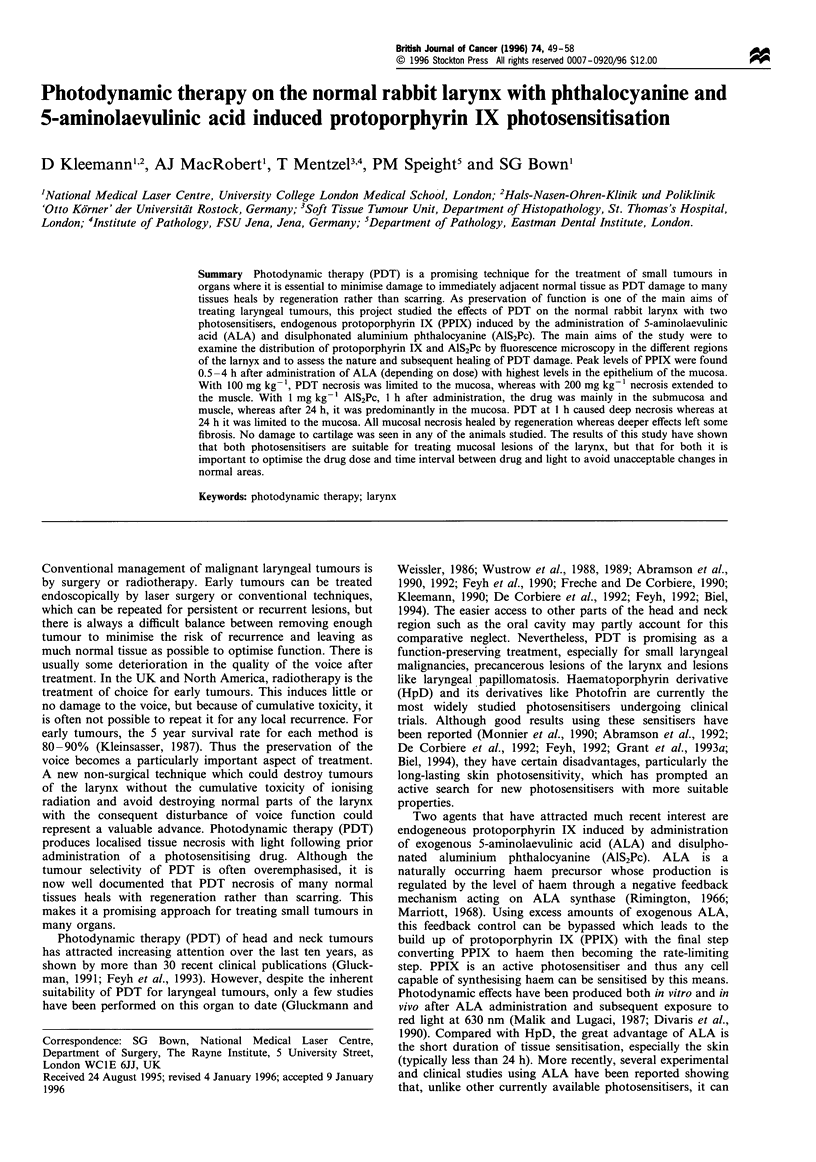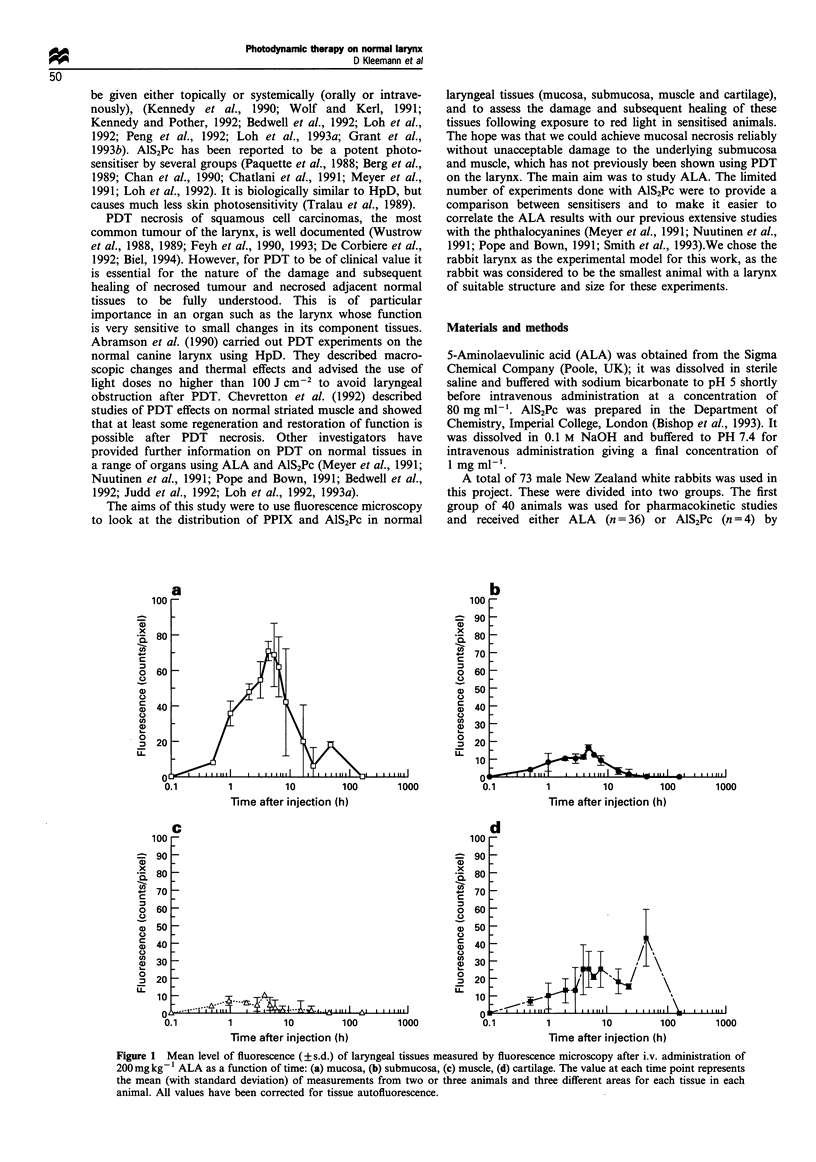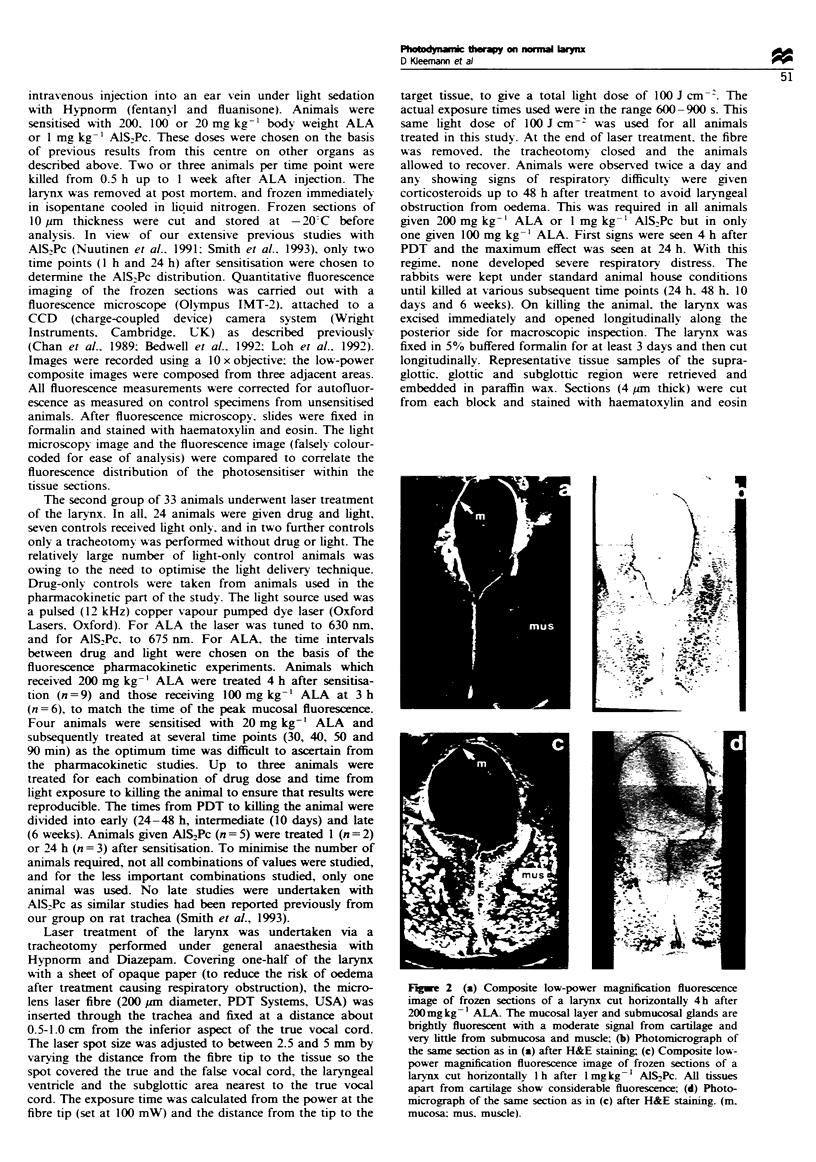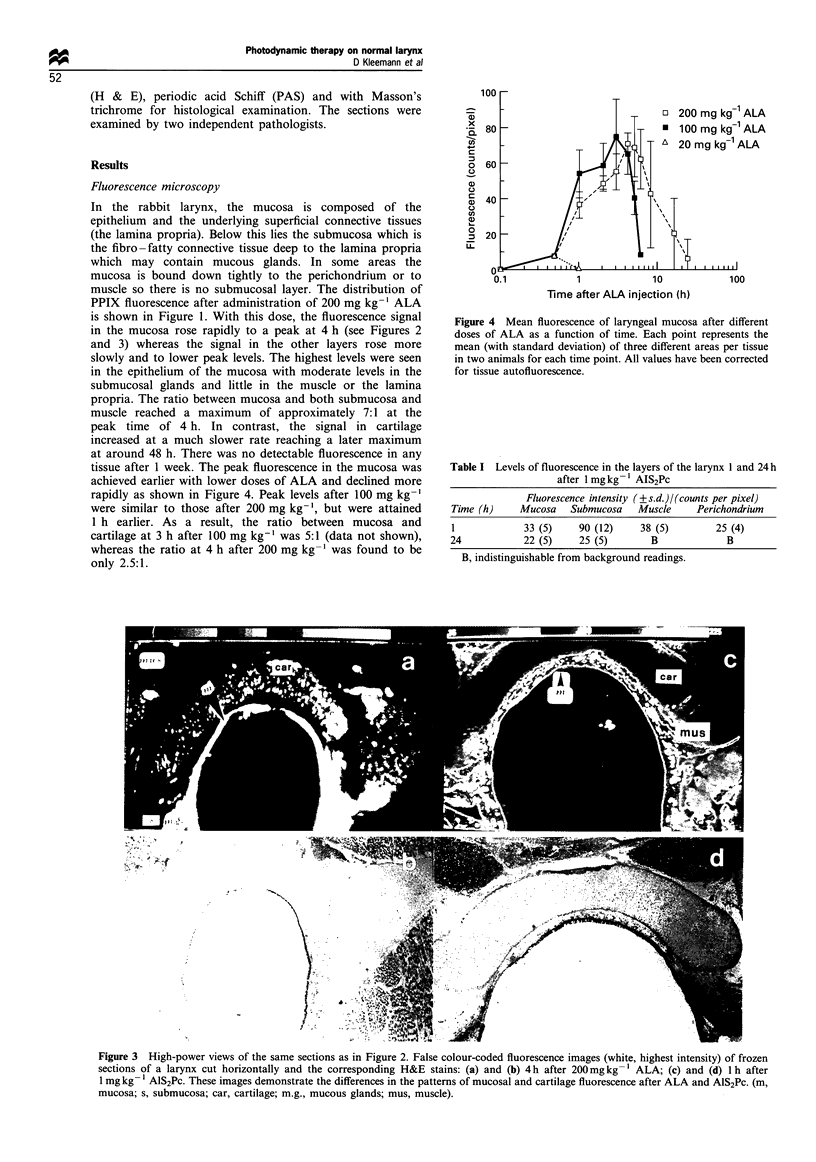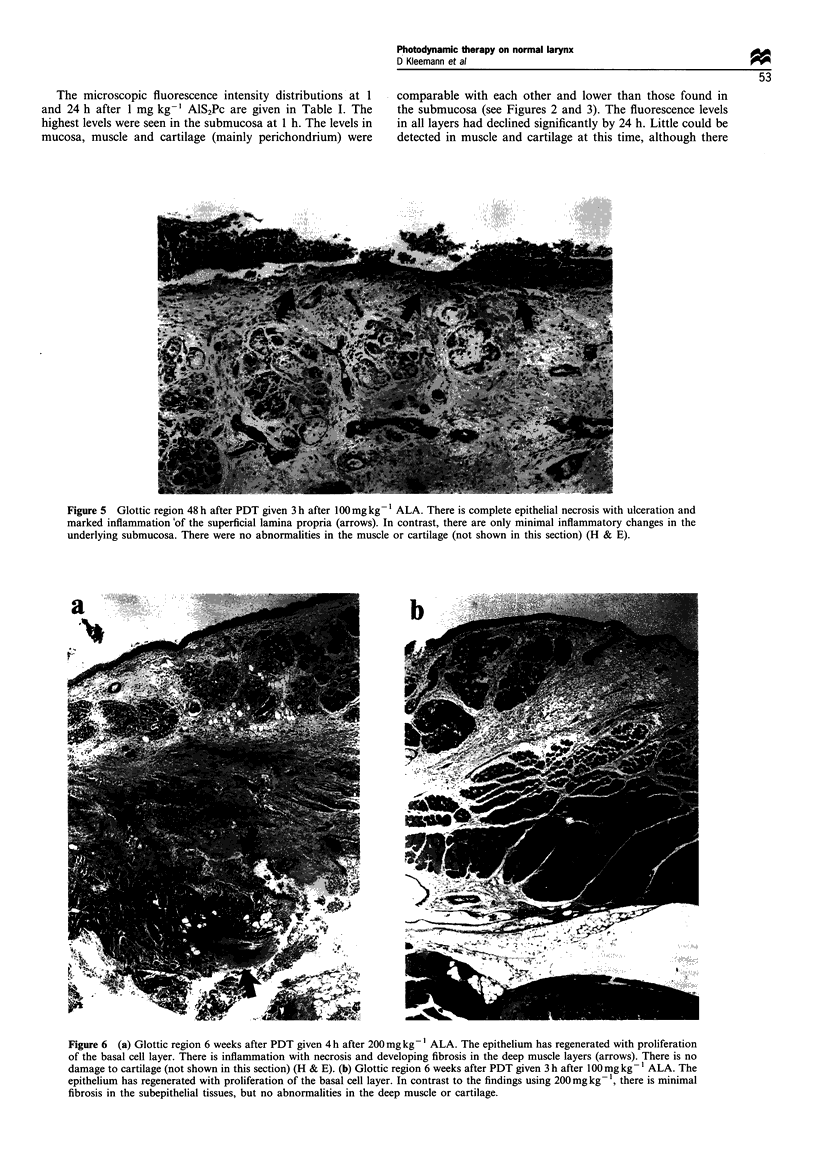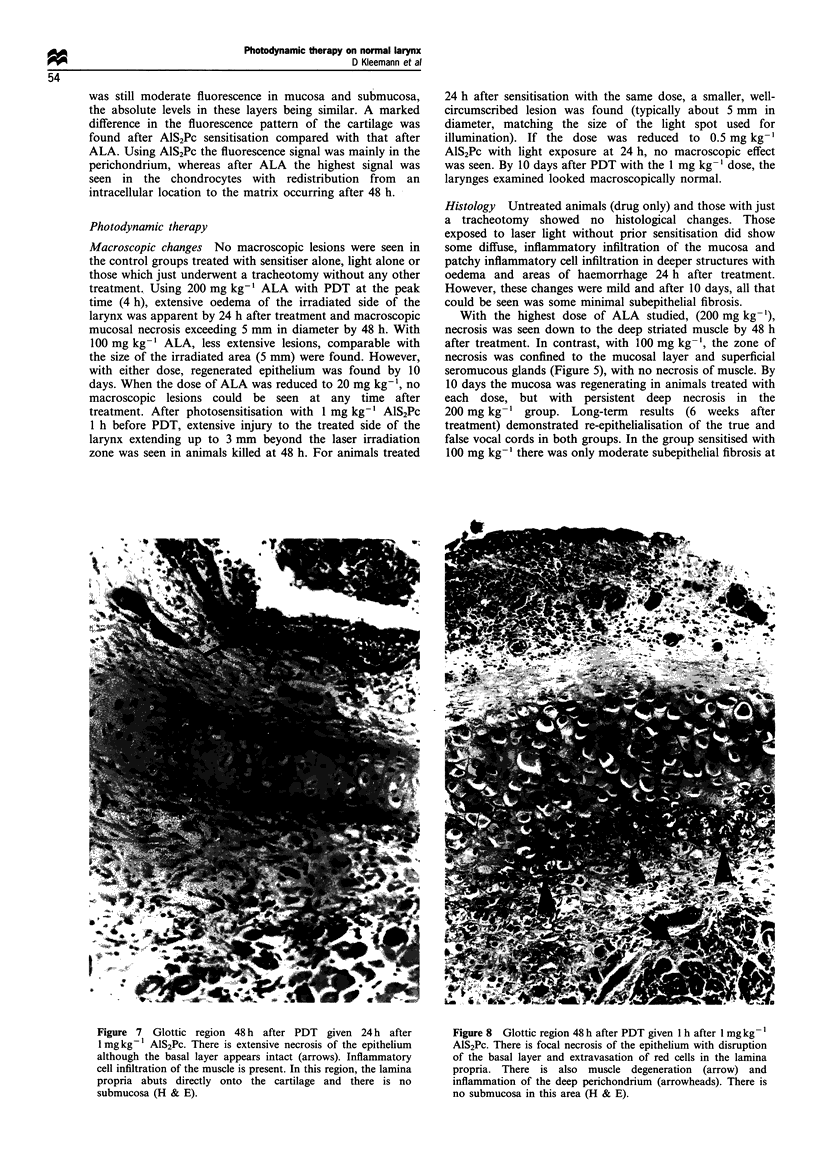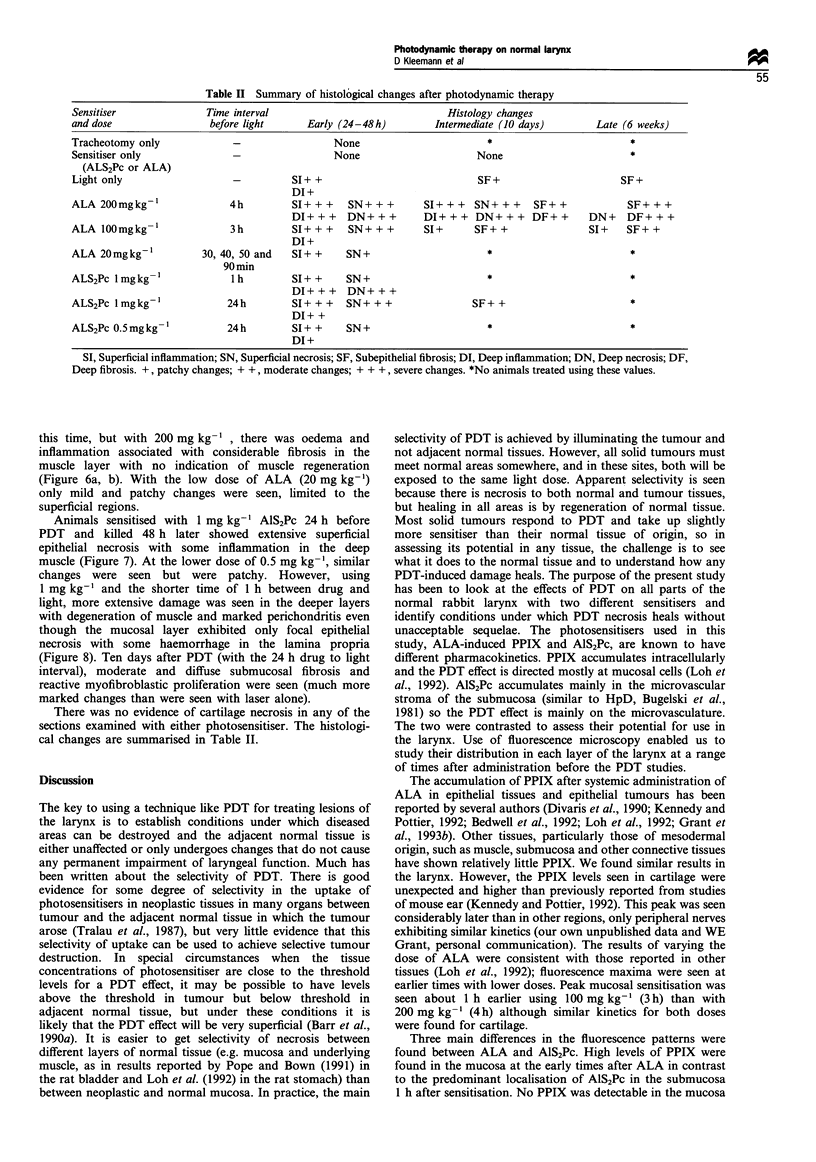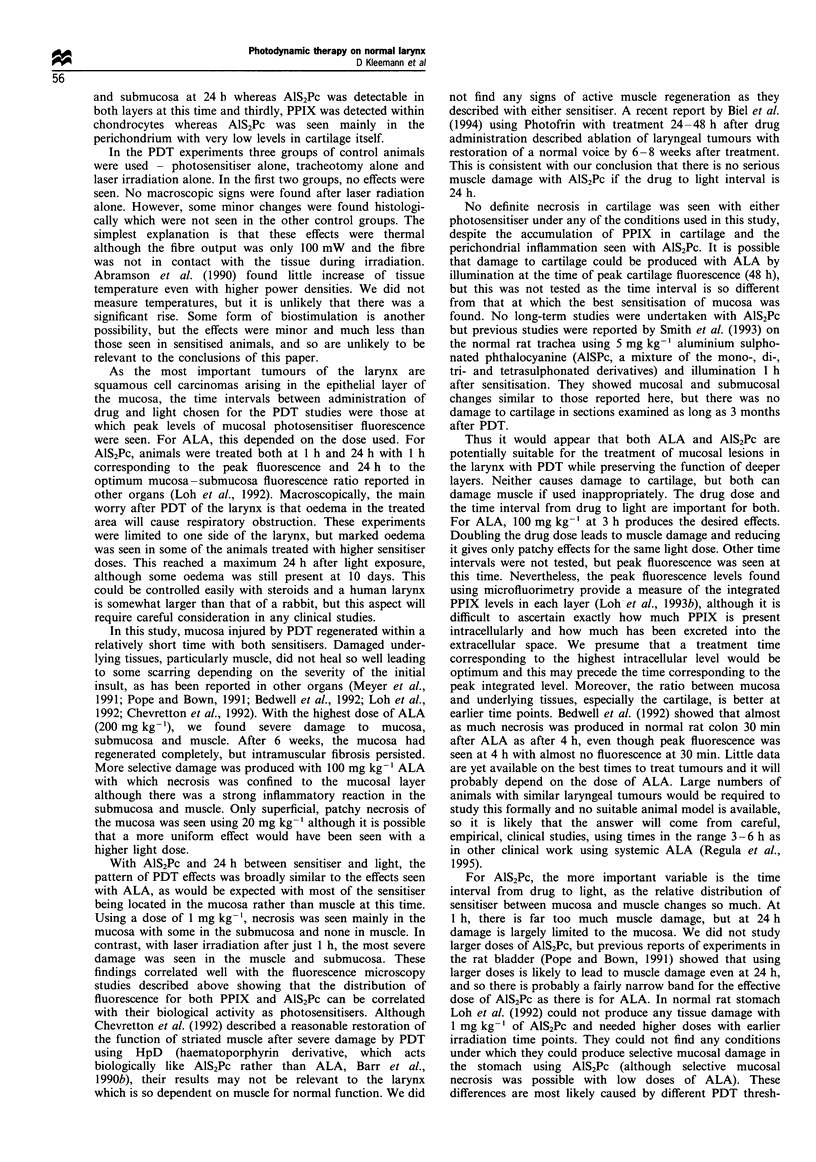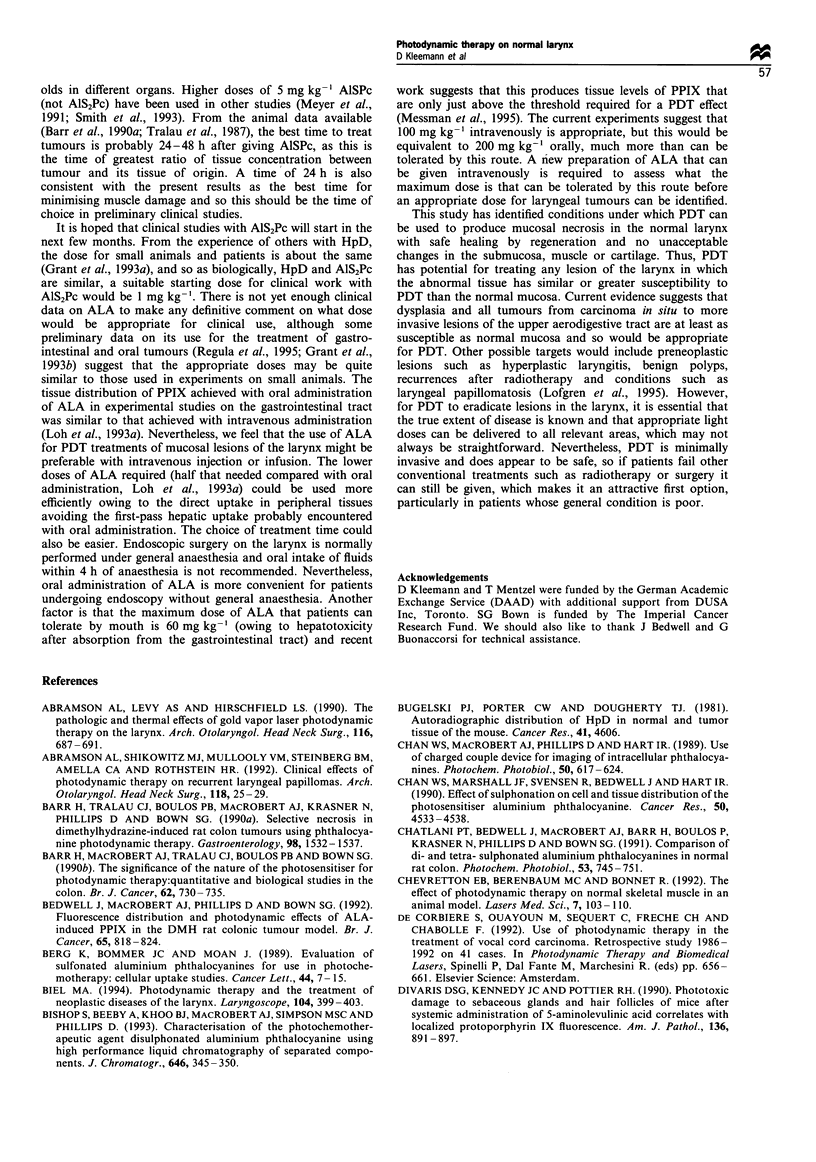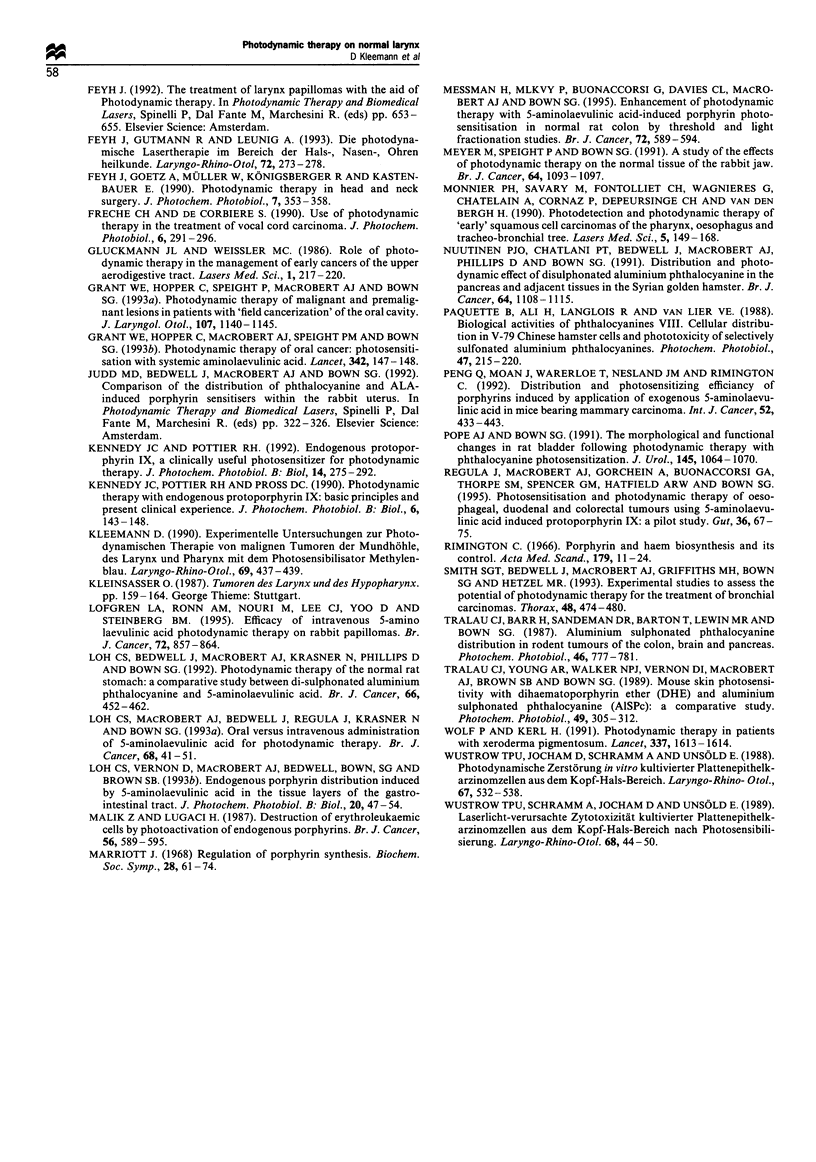# Photodynamic therapy on the normal rabbit larynx with phthalocyanine and 5-aminolaevulinic acid induced protoporphyrin IX photosensitisation.

**DOI:** 10.1038/bjc.1996.314

**Published:** 1996-07

**Authors:** D. Kleemann, A. J. MacRobert, T. Mentzel, P. M. Speight, S. G. Bown

**Affiliations:** National Medical Laser Centre, University College London Medical School.

## Abstract

**Images:**


British Journal of Cancer (1996) 74, 49-58
© 1996 Stockton Press All rights reserved 0007-0920/96 $12.00

### Photodynamic therapy on the normal rabbit larynx with phthalocyanine and 5-aminolaevulinic acid induced protoporphyrin IX photosensitisation

D Kleemann[1,2], AJ MacRobert[1], T Mentzel[3,4], PM Speight[5] and SG Bown[1]

[1]*National Medical Laser Centre, University College London Medical School, London;* [2]*Hals-Nasen-Ohren-Klinik und Poliklinik 'Otto Körner' der Universität Rostock, Germany;* [3]*Soft Tissue Tumour Unit, Department of Histopathology, St. Thomas's Hospital, London;* [4]*Institute of Pathology, FSU Jena, Jena, Germany;* [5]*Department of Pathology, Eastman Dental Institute, London.*

**Summary** Photodynamic therapy (PDT) is a promising technique for the treatment of small tumours in organs where it is essential to minimise damage to immediately adjacent normal tissue as PDT damage to many tissues heals by regeneration rather than scarring. As preservation of function is one of the main aims of treating laryngeal tumours, this project studied the effects of PDT on the normal rabbit larynx with two photosensitisers, endogenous protoporphyrin IX (PPIX) induced by the administration of 5-aminolaevulinic acid (ALA) and disulphonated aluminium phthalocyanine ($AlS_2Pc$). The main aims of the study were to examine the distribution of protoporphyrin IX and $AlS_2Pc$ by fluorescence microscopy in the different regions of the larnyx and to assess the nature and subsequent healing of PDT damage. Peak levels of PPIX were found 0.5–4 h after administration of ALA (depending on dose) with highest levels in the epithelium of the mucosa. With 100 mg $kg^{-1}$, PDT necrosis was limited to the mucosa, whereas with 200 mg $kg^{-1}$ necrosis extended to the muscle. With 1 mg $kg^{-1}$ $AlS_2Pc$, 1 h after administration, the drug was mainly in the submucosa and muscle, whereas after 24 h, it was predominantly in the mucosa. PDT at 1 h caused deep necrosis whereas at 24 h it was limited to the mucosa. All mucosal necrosis healed by regeneration whereas deeper effects left some fibrosis. No damage to cartilage was seen in any of the animals studied. The results of this study have shown that both photosensitisers are suitable for treating mucosal lesions of the larynx, but that for both it is important to optimise the drug dose and time interval between drug and light to avoid unacceptable changes in normal areas.

**Keywords:** photodynamic therapy; larynx

Conventional management of malignant laryngeal tumours is by surgery or radiotherapy. Early tumours can be treated endoscopically by laser surgery or conventional techniques, which can be repeated for persistent or recurrent lesions, but there is always a difficult balance between removing enough tumour to minimise the risk of recurrence and leaving as much normal tissue as possible to optimise function. There is usually some deterioration in the quality of the voice after treatment. In the UK and North America, radiotherapy is the treatment of choice for early tumours. This induces little or no damage to the voice, but because of cumulative toxicity, it is often not possible to repeat it for any local recurrence. For early tumours, the 5 year survival rate for each method is 80–90% (Kleinsasser, 1987). Thus the preservation of the voice becomes a particularly important aspect of treatment. A new non-surgical technique which could destroy tumours of the larynx without the cumulative toxicity of ionising radiation and avoid destroying normal parts of the larynx with the consequent disturbance of voice function could represent a valuable advance. Photodynamic therapy (PDT) produces localised tissue necrosis with light following prior administration of a photosensitising drug. Although the tumour selectivity of PDT is often overemphasised, it is now well documented that PDT necrosis of many normal tissues heals with regeneration rather than scarring. This makes it a promising approach for treating small tumours in many organs.

Photodynamic therapy (PDT) of head and neck tumours has attracted increasing attention over the last ten years, as shown by more than 30 recent clinical publications (Gluckman, 1991; Feyh *et al.*, 1993). However, despite the inherent suitability of PDT for laryngeal tumours, only a few studies have been performed on this organ to date (Gluckmann and Weissler, 1986; Wustrow *et al.*, 1988, 1989; Abramson *et al.*, 1990, 1992; Feyh *et al.*, 1990; Freche and De Corbiere, 1990; Kleemann, 1990; De Corbiere *et al.*, 1992; Feyh, 1992; Biel, 1994). The easier access to other parts of the head and neck region such as the oral cavity may partly account for this comparative neglect. Nevertheless, PDT is promising as a function-preserving treatment, especially for small laryngeal malignancies, precancerous lesions of the larynx and lesions like laryngeal papillomatosis. Haematoporphyrin derivative (HpD) and its derivatives like Photofrin are currently the most widely studied photosensitisers undergoing clinical trials. Although good results using these sensitisers have been reported (Monnier *et al.*, 1990; Abramson *et al.*, 1992; De Corbiere *et al.*, 1992; Feyh, 1992; Grant *et al.*, 1993*a*; Biel, 1994), they have certain disadvantages, particularly the long-lasting skin photosensitivity, which has prompted an active search for new photosensitisers with more suitable properties.

Two agents that have attracted much recent interest are endogeneous protoporphyrin IX induced by administration of exogenous 5-aminolaevulinic acid (ALA) and disulphonated aluminium phthalocyanine ($AlS_2Pc$). ALA is a naturally occurring haem precursor whose production is regulated by the level of haem through a negative feedback mechanism acting on ALA synthase (Rimington, 1966; Marriott, 1968). Using excess amounts of exogenous ALA, this feedback control can be bypassed which leads to the build up of protoporphyrin IX (PPIX) with the final step converting PPIX to haem then becoming the rate-limiting step. PPIX is an active photosensitiser and thus any cell capable of synthesising haem can be sensitised by this means. Photodynamic effects have been produced both *in vitro* and *in vivo* after ALA administration and subsequent exposure to red light at 630 nm (Malik and Lugaci, 1987; Divaris *et al.*, 1990). Compared with HpD, the great advantage of ALA is the short duration of tissue sensitisation, especially the skin (typically less than 24 h). More recently, several experimental and clinical studies using ALA have been reported showing that, unlike other currently available photosensitisers, it can

Correspondence: SG Bown, National Medical Laser Centre, Department of Surgery, The Rayne Institute, 5 University Street, London WC1E 6JJ, UK
Received 24 August 1995; revised 4 January 1996; accepted 9 January 1996

be given either topically or systemically (orally or intravenously), (Kennedy *et al.*, 1990; Wolf and Kerl, 1991; Kennedy and Pother, 1992; Bedwell *et al.*, 1992; Loh *et al.*, 1992; Peng *et al.*, 1992; Loh *et al.*, 1993*a*; Grant *et al.*, 1993*b*). $AlS_2Pc$ has been reported to be a potent photosensitiser by several groups (Paquette *et al.*, 1988; Berg *et al.*, 1989; Chan *et al.*, 1990; Chatlani *et al.*, 1991; Meyer *et al.*, 1991; Loh *et al.*, 1992). It is biologically similar to HpD, but causes much less skin photosensitivity (Tralau *et al.*, 1989).

PDT necrosis of squamous cell carcinomas, the most common tumour of the larynx, is well documented (Wustrow *et al.*, 1988, 1989; Feyh *et al.*, 1990, 1993; De Corbiere *et al.*, 1992; Biel, 1994). However, for PDT to be of clinical value it is essential for the nature of the damage and subsequent healing of necrosed tumour and necrosed adjacent normal tissues to be fully understood. This is of particular importance in an organ such as the larynx whose function is very sensitive to small changes in its component tissues. Abramson *et al.* (1990) carried out PDT experiments on the normal canine larynx using HpD. They described macroscopic changes and thermal effects and advised the use of light doses no higher than 100 J $cm^{-2}$ to avoid laryngeal obstruction after PDT. Chevretton *et al.* (1992) described studies of PDT effects on normal striated muscle and showed that at least some regeneration and restoration of function is possible after PDT necrosis. Other investigators have provided further information on PDT on normal tissues in a range of organs using ALA and $AlS_2Pc$ (Meyer *et al.*, 1991; Nuutinen *et al.*, 1991; Pope and Bown, 1991; Bedwell *et al.*, 1992; Judd *et al.*, 1992; Loh *et al.*, 1992, 1993*a*).

The aims of this study were to use fluorescence microscopy to look at the distribution of PPIX and $AlS_2Pc$ in normal laryngeal tissues (mucosa, submucosa, muscle and cartilage), and to assess the damage and subsequent healing of these tissues following exposure to red light in sensitised animals. The hope was that we could achieve mucosal necrosis reliably without unacceptable damage to the underlying submucosa and muscle, which has not previously been shown using PDT on the larynx. The main aim was to study ALA. The limited number of experiments done with $AlS_2Pc$ were to provide a comparison between sensitisers and to make it easier to correlate the ALA results with our previous extensive studies with the phthalocyanines (Meyer *et al.*, 1991; Nuutinen *et al.*, 1991; Pope and Bown, 1991; Smith *et al.*, 1993).We chose the rabbit larynx as the experimental model for this work, as the rabbit was considered to be the smallest animal with a larynx of suitable structure and size for these experiments.

### Materials and methods

5-Aminolaevulinic acid (ALA) was obtained from the Sigma Chemical Company (Poole, UK); it was dissolved in sterile saline and buffered with sodium bicarbonate to pH 5 shortly before intravenous administration at a concentration of 80 mg $ml^{-1}$. $AlS_2Pc$ was prepared in the Department of Chemistry, Imperial College, London (Bishop *et al.*, 1993). It was dissolved in 0.1 M NaOH and buffered to PH 7.4 for intravenous administration giving a final concentration of 1 mg $ml^{-1}$.

A total of 73 male New Zealand white rabbits was used in this project. These were divided into two groups. The first group of 40 animals was used for pharmacokinetic studies and received either ALA ($n=36$) or $AlS_2Pc$ ($n=4$) by

**Figure 1** Mean level of fluorescence (±s.d.) of laryngeal tissues measured by fluorescence microscopy after i.v. administration of 200 mg $kg^{-1}$ ALA as a function of time: (**a**) mucosa, (**b**) submucosa, (**c**) muscle, (**d**) cartilage. The value at each time point represents the mean (with standard deviation) of measurements from two or three animals and three different areas for each tissue in each animal. All values have been corrected for tissue autofluorescence.

intravenous injection into an ear vein under light sedation with Hypnorm (fentanyl and fluanisone). Animals were sensitised with 200, 100 or 20 mg $kg^{-1}$ body weight ALA or 1 mg $kg^{-1}$ $AlS_2Pc$. These doses were chosen on the basis of previous results from this centre on other organs as described above. Two or three animals per time point were killed from 0.5 h up to 1 week after ALA injection. The larynx was removed at post mortem, and frozen immediately in isopentane cooled in liquid nitrogen. Frozen sections of 10 $\mu$m thickness were cut and stored at $-20^{\circ}$C before analysis. In view of our extensive previous studies with $AlS_2Pc$ (Nuutinen *et al.*, 1991; Smith *et al.*, 1993), only two time points (1 h and 24 h) after sensitisation were chosen to determine the $AlS_2Pc$ distribution. Quantitative fluorescence imaging of the frozen sections was carried out with a fluorescence microscope (Olympus IMT-2), attached to a CCD (charge-coupled device) camera system (Wright Instruments, Cambridge, UK) as described previously (Chan *et al.*, 1989; Bedwell *et al.*, 1992; Loh *et al.*, 1992). Images were recorded using a 10 × objective; the low-power composite images were composed from three adjacent areas. All fluorescence measurements were corrected for autofluorescence as measured on control specimens from unsensitised animals. After fluorescence microscopy, slides were fixed in formalin and stained with haematoxylin and eosin. The light microscopy image and the fluorescence image (falsely colour-coded for ease of analysis) were compared to correlate the fluorescence distribution of the photosensitiser within the tissue sections.

The second group of 33 animals underwent laser treatment of the larynx. In all, 24 animals were given drug and light, seven controls received light only, and in two further controls only a tracheotomy was performed without drug or light. The relatively large number of light-only control animals was owing to the need to optimise the light delivery technique. Drug-only controls were taken from animals used in the pharmacokinetic part of the study. The light source used was a pulsed (12 kHz) copper vapour pumped dye laser (Oxford Lasers, Oxford). For ALA the laser was tuned to 630 nm, and for $AlS_2Pc$, to 675 nm. For ALA, the time intervals between drug and light were chosen on the basis of the fluorescence pharmacokinetic experiments. Animals which received 200 mg $kg^{-1}$ ALA were treated 4 h after sensitisation ($n=9$) and those receiving 100 mg $kg^{-1}$ ALA at 3 h ($n=6$), to match the time of the peak mucosal fluorescence. Four animals were sensitised with 20 mg $kg^{-1}$ ALA and subsequently treated at several time points (30, 40, 50 and 90 min) as the optimum time was difficult to ascertain from the pharmacokinetic studies. Up to three animals were treated for each combination of drug dose and time from light exposure to killing the animal to ensure that results were reproducible. The times from PDT to killing the animal were divided into early (24–48 h, intermediate (10 days) and late (6 weeks). Animals given $AlS_2Pc$ ($n=5$) were treated 1 ($n=2$) or 24 h ($n=3$) after sensitisation. To minimise the number of animals required, not all combinations of values were studied, and for the less important combinations studied, only one animal was used. No late studies were undertaken with $AlS_2Pc$ as similar studies had been reported previously from our group on rat trachea (Smith *et al.*, 1993).

Laser treatment of the larynx was undertaken via a tracheotomy performed under general anaesthesia with Hypnorm and Diazepam. Covering one-half of the larynx with a sheet of opaque paper (to reduce the risk of oedema after treatment causing respiratory obstruction), the microlens laser fibre (200 $\mu$m diameter, PDT Systems, USA) was inserted through the trachea and fixed at a distance about 0.5-1.0 cm from the inferior aspect of the true vocal cord. The laser spot size was adjusted to between 2.5 and 5 mm by varying the distance from the fibre tip to the tissue so the spot covered the true and the false vocal cord, the laryngeal ventricle and the subglottic area nearest to the true vocal cord. The exposure time was calculated from the power at the fibre tip (set at 100 mW) and the distance from the tip to the target tissue, to give a total light dose of 100 J $cm^{-2}$. The actual exposure times used were in the range 600–900 s. This same light dose of 100 J $cm^{-2}$ was used for all animals treated in this study. At the end of laser treatment, the fibre was removed, the tracheotomy closed and the animals allowed to recover. Animals were observed twice a day and any showing signs of respiratory difficulty were given corticosteroids up to 48 h after treatment to avoid laryngeal obstruction from oedema. This was required in all animals given 200 mg $kg^{-1}$ ALA or 1 mg $kg^{-1}$ $AlS_2Pc$ but in only one given 100 mg $kg^{-1}$ ALA. First signs were seen 4 h after PDT and the maximum effect was seen at 24 h. With this regime, none developed severe respiratory distress. The rabbits were kept under standard animal house conditions until killed at various subsequent time points (24 h, 48 h, 10 days and 6 weeks). On killing the animal, the larynx was excised immediately and opened longitudinally along the posterior side for macroscopic inspection. The larynx was fixed in 5% buffered formalin for at least 3 days and then cut longitudinally. Representative tissue samples of the supraglottic, glottic and subglottic region were retrieved and embedded in paraffin wax. Sections (4 $\mu$m thick) were cut from each block and stained with haematoxylin and eosin

**Figure 2** (**a**) Composite low-power magnification fluorescence image of frozen sections of a larynx cut horizontally 4 h after 200 mg $kg^{-1}$ ALA. The mucosal layer and submucosal glands are brightly fluorescent with a moderate signal from cartilage and very little from submucosa and muscle; (**b**) Photomicrograph of the same section as in (**a**) after H&E staining; (**c**) Composite low-power magnification fluorescence image of frozen sections of a larynx cut horizontally 1 h after 1 mg $kg^{-1}$ $AlS_2Pc$. All tissues apart from cartilage show considerable fluorescence; (**d**) Photomicrograph of the same section as in (**c**) after H&E staining. (m, mucosa; mus, muscle).

(H & E), periodic acid Schiff (PAS) and with Masson's trichrome for histological examination. The sections were examined by two independent pathologists.

### Results

### *Fluorescence microscopy*

In the rabbit larynx, the mucosa is composed of the epithelium and the underlying superficial connective tissues (the lamina propria). Below this lies the submucosa which is the fibro-fatty connective tissue deep to the lamina propria which may contain mucous glands. In some areas the mucosa is bound down tightly to the perichondrium or to muscle so there is no submucosal layer. The distribution of PPIX fluorescence after administration of 200 mg $kg^{-1}$ ALA is shown in Figure 1. With this dose, the fluorescence signal in the mucosa rose rapidly to a peak at 4 h (see Figures 2 and 3) whereas the signal in the other layers rose more slowly and to lower peak levels. The highest levels were seen in the epithelium of the mucosa with moderate levels in the submucosal glands and little in the muscle or the lamina propria. The ratio between mucosa and both submucosa and muscle reached a maximum of approximately 7:1 at the peak time of 4 h. In contrast, the signal in cartilage increased at a much slower rate reaching a later maximum at around 48 h. There was no detectable fluorescence in any tissue after 1 week. The peak fluorescence in the mucosa was achieved earlier with lower doses of ALA and declined more rapidly as shown in Figure 4. Peak levels after 100 mg $kg^{-1}$ were similar to those after 200 mg $kg^{-1}$, but were attained 1 h earlier. As a result, the ratio between mucosa and cartilage at 3 h after 100 mg $kg^{-1}$ was 5:1 (data not shown), whereas the ratio at 4 h after 200 mg $kg^{-1}$ was found to be only 2.5:1.

**Figure 4** Mean fluorescence of laryngeal mucosa after different doses of ALA as a function of time. Each point represents the mean (with standard deviation) of three different areas per tissue in two animals for each time point. All values have been corrected for tissue autofluorescence.

**Table I** Levels of fluorescence in the layers of the larynx 1 and 24 h after 1 mg $kg^{-1}$ $AlS_2Pc$

| *Time (h)* | *Fluorescence intensity (±s.d.)/(counts per pixel)* Mucosa | Submucosa | Muscle | Perichondrium |
|---|---|---|---|---|
| 1 | 33 (5) | 90 (12) | 38 (5) | 25 (4) |
| 24 | 22 (5) | 25 (5) | B | B |

B, indistinguishable from background readings.

**Figure 3** High-power views of the same sections as in Figure 2. False colour-coded fluorescence images (white, highest intensity) of frozen sections of a larynx cut horizontally and the corresponding H&E stains: (**a**) and (**b**) 4 h after 200 mg $kg^{-1}$ ALA; (**c**) and (**d**) 1 h after 1 mg $kg^{-1}$ $AlS_2Pc$. These images demonstrate the differences in the patterns of mucosal and cartilage fluorescence after ALA and $AlS_2Pc$. (m, mucosa; s, submucosa; car, cartilage; m.g., mucous glands; mus, muscle).

The microscopic fluorescence intensity distributions at 1 and 24 h after 1 mg $kg^{-1}$ $AlS_2Pc$ are given in Table I. The highest levels were seen in the submucosa at 1 h. The levels in mucosa, muscle and cartilage (mainly perichondrium) were comparable with each other and lower than those found in the submucosa (see Figures 2 and 3). The fluorescence levels in all layers had declined significantly by 24 h. Little could be detected in muscle and cartilage at this time, although there

**Figure 5** Glottic region 48 h after PDT given 3 h after 100 mg $kg^{-1}$ ALA. There is complete epithelial necrosis with ulceration and marked inflammation of the superficial lamina propria (arrows). In contrast, there are only minimal inflammatory changes in the underlying submucosa. There were no abnormalities in the muscle or cartilage (not shown in this section) (H & E).

**Figure 6** (**a**) Glottic region 6 weeks after PDT given 4 h after 200 mg $kg^{-1}$ ALA. The epithelium has regenerated with proliferation of the basal cell layer. There is inflammation with necrosis and developing fibrosis in the deep muscle layers (arrows). There is no damage to cartilage (not shown in this section) (H & E). (**b**) Glottic region 6 weeks after PDT given 3 h after 100 mg $kg^{-1}$ ALA. The epithelium has regenerated with proliferation of the basal cell layer. In contrast to the findings using 200 mg $kg^{-1}$, there is minimal fibrosis in the subepithelial tissues, but no abnormalities in the deep muscle or cartilage.

was still moderate fluorescence in mucosa and submucosa, the absolute levels in these layers being similar. A marked difference in the fluorescence pattern of the cartilage was found after $AlS_2Pc$ sensitisation compared with that after ALA. Using $AlS_2Pc$ the fluorescence signal was mainly in the perichondrium, whereas after ALA the highest signal was seen in the chondrocytes with redistribution from an intracellular location to the matrix occurring after 48 h.

*Photodynamic therapy*

*Macroscopic changes* No macroscopic lesions were seen in the control groups treated with sensitiser alone, light alone or those which just underwent a tracheotomy without any other treatment. Using 200 mg $kg^{-1}$ ALA with PDT at the peak time (4 h), extensive oedema of the irradiated side of the larynx was apparent by 24 h after treatment and macroscopic mucosal necrosis exceeding 5 mm in diameter by 48 h. With 100 mg $kg^{-1}$ ALA, less extensive lesions, comparable with the size of the irradiated area (5 mm) were found. However, with either dose, regenerated epithelium was found by 10 days. When the dose of ALA was reduced to 20 mg $kg^{-1}$, no macroscopic lesions could be seen at any time after treatment. After photosensitisation with 1 mg $kg^{-1}$ $AlS_2Pc$ 1 h before PDT, extensive injury to the treated side of the larynx extending up to 3 mm beyond the laser irradiation zone was seen in animals killed at 48 h. For animals treated 24 h after sensitisation with the same dose, a smaller, well-circumscribed lesion was found (typically about 5 mm in diameter, matching the size of the light spot used for illumination). If the dose was reduced to 0.5 mg $kg^{-1}$ $AlS_2Pc$ with light exposure at 24 h, no macroscopic effect was seen. By 10 days after PDT with the 1 mg $kg^{-1}$ dose, the larynges examined looked macroscopically normal.

*Histology* Untreated animals (drug only) and those with just a tracheotomy showed no histological changes. Those exposed to laser light without prior sensitisation did show some diffuse, inflammatory infiltration of the mucosa and patchy inflammatory cell infiltration in deeper structures with oedema and areas of haemorrhage 24 h after treatment. However, these changes were mild and after 10 days, all that could be seen was some minimal subepithelial fibrosis.

With the highest dose of ALA studied, (200 mg $kg^{-1}$), necrosis was seen down to the deep striated muscle by 48 h after treatment. In contrast, with 100 mg $kg^{-1}$, the zone of necrosis was confined to the mucosal layer and superficial seromucous glands (Figure 5), with no necrosis of muscle. By 10 days the mucosa was regenerating in animals treated with each dose, but with persistent deep necrosis in the 200 mg $kg^{-1}$ group. Long-term results (6 weeks after treatment) demonstrated re-epithelialisation of the true and false vocal cords in both groups. In the group sensitised with 100 mg $kg^{-1}$ there was only moderate subepithelial fibrosis at

**Figure 7** Glottic region 48 h after PDT given 24 h after 1 mg $kg^{-1}$ $AlS_2Pc$. There is extensive necrosis of the epithelium although the basal layer appears intact (arrows). Inflammatory cell infiltration of the muscle is present. In this region, the lamina propria abuts directly onto the cartilage and there is no submucosa (H & E).

**Figure 8** Glottic region 48 h after PDT given 1 h after 1 mg $kg^{-1}$ $AlS_2Pc$. There is focal necrosis of the epithelium with disruption of the basal layer and extravasation of red cells in the lamina propria. There is also muscle degeneration (arrow) and inflammation of the deep perichondrium (arrowheads). There is no submucosa in this area (H & E).

**Table II** Summary of histological changes after photodynamic therapy

| *Sensitiser and dose* | *Time interval before light* | *Histology changes* Early (24–48 h) | *Intermediate (10 days)* | *Late (6 weeks)* |
|---|---|---|---|---|
| Tracheotomy only | – | None | * | * |
| Sensitiser only ($ALS_2Pc$ or ALA) | – | None | None | * |
| Light only | – | SI+ +<br>DI+ | SF+ | SF+ |
| ALA 200 mg $kg^{-1}$ | 4 h | SI+ + + SN+ + +<br>DI+ + + DN+ + + | SI+ + + SN+ + + SF+ +<br>DI+ + + DN+ + + DF+ + | SF+ + +<br>DN+ DF+ + + |
| ALA 100 mg $kg^{-1}$ | 3 h | SI+ + + SN+ + +<br>DI+ | SI+ SF+ + | SI+ SF+ + |
| ALA 20 mg $kg^{-1}$ | 30, 40, 50 and 90 min | SI+ + SN+ | * | * |
| $ALS_2Pc$ 1 mg $kg^{-1}$ | 1 h | SI+ + SN+<br>DI+ + + DN+ + + | * | * |
| $ALS_2Pc$ 1 mg $kg^{-1}$ | 24 h | SI+ + + SN+ + +<br>DI+ + | SF+ + | * |
| $ALS_2Pc$ 0.5 mg $kg^{-1}$ | 24 h | SI+ + SN+<br>DI+ | * | * |

SI, Superficial inflammation; SN, Superficial necrosis; SF, Subepithelial fibrosis; DI, Deep inflammation; DN, Deep necrosis; DF, Deep fibrosis. +, patchy changes; + +, moderate changes; + + +, severe changes. *No animals treated using these values.

this time, but with 200 mg $kg^{-1}$ , there was oedema and inflammation associated with considerable fibrosis in the muscle layer with no indication of muscle regeneration (Figure 6a, b). With the low dose of ALA (20 mg $kg^{-1}$) only mild and patchy changes were seen, limited to the superficial regions.

Animals sensitised with 1 mg $kg^{-1}$ $AlS_2Pc$ 24 h before PDT and killed 48 h later showed extensive superficial epithelial necrosis with some inflammation in the deep muscle (Figure 7). At the lower dose of 0.5 mg $kg^{-1}$, similar changes were seen but were patchy. However, using 1 mg $kg^{-1}$ and the shorter time of 1 h between drug and light, more extensive damage was seen in the deeper layers with degeneration of muscle and marked perichondritis even though the mucosal layer exhibited only focal epithelial necrosis with some haemorrhage in the lamina propria (Figure 8). Ten days after PDT (with the 24 h drug to light interval), moderate and diffuse submucosal fibrosis and reactive myofibroblastic proliferation were seen (much more marked changes than were seen with laser alone).

There was no evidence of cartilage necrosis in any of the sections examined with either photosensitiser. The histological changes are summarised in Table II.

### Discussion

The key to using a technique like PDT for treating lesions of the larynx is to establish conditions under which diseased areas can be destroyed and the adjacent normal tissue is either unaffected or only undergoes changes that do not cause any permanent impairment of laryngeal function. Much has been written about the selectivity of PDT. There is good evidence for some degree of selectivity in the uptake of photosensitisers in neoplastic tissues in many organs between tumour and the adjacent normal tissue in which the tumour arose (Tralau *et al.*, 1987), but very little evidence that this selectivity of uptake can be used to achieve selective tumour destruction. In special circumstances when the tissue concentrations of photosensitiser are close to the threshold levels for a PDT effect, it may be possible to have levels above the threshold in tumour but below threshold in adjacent normal tissue, but under these conditions it is likely that the PDT effect will be very superficial (Barr *et al.*, 1990*a*). It is easier to get selectivity of necrosis between different layers of normal tissue (e.g. mucosa and underlying muscle, as in results reported by Pope and Bown (1991) in the rat bladder and Loh *et al.* (1992) in the rat stomach) than between neoplastic and normal mucosa. In practice, the main selectivity of PDT is achieved by illuminating the tumour and not adjacent normal tissues. However, all solid tumours must meet normal areas somewhere, and in these sites, both will be exposed to the same light dose. Apparent selectivity is seen because there is necrosis to both normal and tumour tissues, but healing in all areas is by regeneration of normal tissue. Most solid tumours respond to PDT and take up slightly more sensitiser than their normal tissue of origin, so in assessing its potential in any tissue, the challenge is to see what it does to the normal tissue and to understand how any PDT-induced damage heals. The purpose of the present study has been to look at the effects of PDT on all parts of the normal rabbit larynx with two different sensitisers and identify conditions under which PDT necrosis heals without unacceptable sequelae. The photosensitisers used in this study, ALA-induced PPIX and $AlS_2Pc$, are known to have different pharmacokinetics. PPIX accumulates intracellularly and the PDT effect is directed mostly at mucosal cells (Loh *et al.*, 1992). $AlS_2Pc$ accumulates mainly in the microvascular stroma of the submucosa (similar to HpD, Bugelski *et al.*, 1981) so the PDT effect is mainly on the microvasculature. The two were contrasted to assess their potential for use in the larynx. Use of fluorescence microscopy enabled us to study their distribution in each layer of the larynx at a range of times after administration before the PDT studies.

The accumulation of PPIX after systemic administration of ALA in epithelial tissues and epithelial tumours has been reported by several authors (Divaris *et al.*, 1990; Kennedy and Pottier, 1992; Bedwell *et al.*, 1992; Loh *et al.*, 1992; Grant *et al.*, 1993*b*). Other tissues, particularly those of mesodermal origin, such as muscle, submucosa and other connective tissues have shown relatively little PPIX. We found similar results in the larynx. However, the PPIX levels seen in cartilage were unexpected and higher than previously reported from studies of mouse ear (Kennedy and Pottier, 1992). This peak was seen considerably later than in other regions, only peripheral nerves exhibiting similar kinetics (our own unpublished data and WE Grant, personal communication). The results of varying the dose of ALA were consistent with those reported in other tissues (Loh *et al.*, 1992); fluorescence maxima were seen at earlier times with lower doses. Peak mucosal sensitisation was seen about 1 h earlier using 100 mg $kg^{-1}$ (3 h) than with 200 mg $kg^{-1}$ (4 h) although similar kinetics for both doses were found for cartilage.

Three main differences in the fluorescence patterns were found between ALA and $AlS_2Pc$. High levels of PPIX were found in the mucosa at the early times after ALA in contrast to the predominant localisation of $AlS_2Pc$ in the submucosa 1 h after sensitisation. No PPIX was detectable in the mucosa and submucosa at 24 h whereas $AlS_2Pc$ was detectable in both layers at this time and thirdly, PPIX was detected within chondrocytes whereas $AlS_2Pc$ was seen mainly in the perichondrium with very low levels in cartilage itself.

In the PDT experiments three groups of control animals were used – photosensitiser alone, tracheotomy alone and laser irradiation alone. In the first two groups, no effects were seen. No macroscopic signs were found after laser radiation alone. However, some minor changes were found histologically which were not seen in the other control groups. The simplest explanation is that these effects were thermal although the fibre output was only 100 mW and the fibre was not in contact with the tissue during irradiation. Abramson *et al.* (1990) found little increase of tissue temperature even with higher power densities. We did not measure temperatures, but it is unlikely that there was a significant rise. Some form of biostimulation is another possibility, but the effects were minor and much less than those seen in sensitised animals, and so are unlikely to be relevant to the conclusions of this paper.

As the most important tumours of the larynx are squamous cell carcinomas arising in the epithelial layer of the mucosa, the time intervals between administration of drug and light chosen for the PDT studies were those at which peak levels of mucosal photosensitiser fluorescence were seen. For ALA, this depended on the dose used. For $AlS_2Pc$, animals were treated both at 1 h and 24 h with 1 h corresponding to the peak fluorescence and 24 h to the optimum mucosa–submucosa fluorescence ratio reported in other organs (Loh *et al.*, 1992). Macroscopically, the main worry after PDT of the larynx is that oedema in the treated area will cause respiratory obstruction. These experiments were limited to one side of the larynx, but marked oedema was seen in some of the animals treated with higher sensitiser doses. This reached a maximum 24 h after light exposure, although some oedema was still present at 10 days. This could be controlled easily with steroids and a human larynx is somewhat larger than that of a rabbit, but this aspect will require careful consideration in any clinical studies.

In this study, mucosa injured by PDT regenerated within a relatively short time with both sensitisers. Damaged underlying tissues, particularly muscle, did not heal so well leading to some scarring depending on the severity of the initial insult, as has been reported in other organs (Meyer *et al.*, 1991; Pope and Bown, 1991; Bedwell *et al.*, 1992; Loh *et al.*, 1992; Chevretton *et al.*, 1992). With the highest dose of ALA (200 mg $kg^{-1}$), we found severe damage to mucosa, submucosa and muscle. After 6 weeks, the mucosa had regenerated completely, but intramuscular fibrosis persisted. More selective damage was produced with 100 mg $kg^{-1}$ ALA with which necrosis was confined to the mucosal layer although there was a strong inflammatory reaction in the submucosa and muscle. Only superficial, patchy necrosis of the mucosa was seen using 20 mg $kg^{-1}$ although it is possible that a more uniform effect would have been seen with a higher light dose.

With $AlS_2Pc$ and 24 h between sensitiser and light, the pattern of PDT effects was broadly similar to the effects seen with ALA, as would be expected with most of the sensitiser being located in the mucosa rather than muscle at this time. Using a dose of 1 mg $kg^{-1}$, necrosis was seen mainly in the mucosa with some in the submucosa and none in muscle. In contrast, with laser irradiation after just 1 h, the most severe damage was seen in the muscle and submucosa. These findings correlated well with the fluorescence microscopy studies described above showing that the distribution of fluorescence for both PPIX and $AlS_2Pc$ can be correlated with their biological activity as photosensitisers. Although Chevretton *et al.* (1992) described a reasonable restoration of the function of striated muscle after severe damage by PDT using HpD (haematoporphyrin derivative, which acts biologically like $AlS_2Pc$ rather than ALA, Barr *et al.*, 1990*b*), their results may not be relevant to the larynx which is so dependent on muscle for normal function. We did not find any signs of active muscle regeneration as they described with either sensitiser. A recent report by Biel *et al.* (1994) using Photofrin with treatment 24–48 h after drug administration described ablation of laryngeal tumours with restoration of a normal voice by 6–8 weeks after treatment. This is consistent with our conclusion that there is no serious muscle damage with $AlS_2Pc$ if the drug to light interval is 24 h.

No definite necrosis in cartilage was seen with either photosensitiser under any of the conditions used in this study, despite the accumulation of PPIX in cartilage and the perichondrial inflammation seen with $AlS_2Pc$. It is possible that damage to cartilage could be produced with ALA by illumination at the time of peak cartilage fluorescence (48 h), but this was not tested as the time interval is so different from that at which the best sensitisation of mucosa was found. No long-term studies were undertaken with $AlS_2Pc$ but previous studies were reported by Smith *et al.* (1993) on the normal rat trachea using 5 mg $kg^{-1}$ aluminium sulphonated phthalocyanine (AlSPc, a mixture of the mono-, di-, tri- and tetrasulphonated derivatives) and illumination 1 h after sensitisation. They showed mucosal and submucosal changes similar to those reported here, but there was no damage to cartilage in sections examined as long as 3 months after PDT.

Thus it would appear that both ALA and $AlS_2Pc$ are potentially suitable for the treatment of mucosal lesions in the larynx with PDT while preserving the function of deeper layers. Neither causes damage to cartilage, but both can damage muscle if used inappropriately. The drug dose and the time interval from drug to light are important for both. For ALA, 100 mg $kg^{-1}$ at 3 h produces the desired effects. Doubling the drug dose leads to muscle damage and reducing it gives only patchy effects for the same light dose. Other time intervals were not tested, but peak fluorescence was seen at this time. Nevertheless, the peak fluorescence levels found using microfluorimetry provide a measure of the integrated PPIX levels in each layer (Loh *et al.*, 1993*b*), although it is difficult to ascertain exactly how much PPIX is present intracellularly and how much has been excreted into the extracellular space. We presume that a treatment time corresponding to the highest intracellular level would be optimum and this may precede the time corresponding to the peak integrated level. Moreover, the ratio between mucosa and underlying tissues, especially the cartilage, is better at earlier time points. Bedwell *et al.* (1992) showed that almost as much necrosis was produced in normal rat colon 30 min after ALA as after 4 h, even though peak fluorescence was seen at 4 h with almost no fluorescence at 30 min. Little data are yet available on the best times to treat tumours and it will probably depend on the dose of ALA. Large numbers of animals with similar laryngeal tumours would be required to study this formally and no suitable animal model is available, so it is likely that the answer will come from careful, empirical, clinical studies, using times in the range 3–6 h as in other clinical work using systemic ALA (Regula *et al.*, 1995).

For $AlS_2Pc$, the more important variable is the time interval from drug to light, as the relative distribution of sensitiser between mucosa and muscle changes so much. At 1 h, there is far too much muscle damage, but at 24 h damage is largely limited to the mucosa. We did not study larger doses of $AlS_2Pc$, but previous reports of experiments in the rat bladder (Pope and Bown, 1991) showed that using larger doses is likely to lead to muscle damage even at 24 h, and so there is probably a fairly narrow band for the effective dose of $AlS_2Pc$ as there is for ALA. In normal rat stomach Loh *et al.* (1992) could not produce any tissue damage with 1 mg $kg^{-1}$ of $AlS_2Pc$ and needed higher doses with earlier irradiation time points. They could not find any conditions under which they could produce selective mucosal damage in the stomach using $AlS_2Pc$ (although selective mucosal necrosis was possible with low doses of ALA). These differences are most likely caused by different PDT thresholds in different organs. Higher doses of 5 mg $kg^{-1}$ AlSPc (not $AlS_2Pc$) have been used in other studies (Meyer *et al.*, 1991; Smith *et al.*, 1993). From the animal data available (Barr *et al.*, 1990*a*; Tralau *et al.*, 1987), the best time to treat tumours is probably 24–48 h after giving AlSPc, as this is the time of greatest ratio of tissue concentration between tumour and its tissue of origin. A time of 24 h is also consistent with the present results as the best time for minimising muscle damage and so this should be the time of choice in preliminary clinical studies.

It is hoped that clinical studies with $AlS_2Pc$ will start in the next few months. From the experience of others with HpD, the dose for small animals and patients is about the same (Grant *et al.*, 1993*a*), and so as biologically, HpD and $AlS_2Pc$ are similar, a suitable starting dose for clinical work with $AlS_2Pc$ would be 1 mg $kg^{-1}$. There is not yet enough clinical data on ALA to make any definitive comment on what dose would be appropriate for clinical use, although some preliminary data on its use for the treatment of gastro-intestinal and oral tumours (Regula *et al.*, 1995; Grant *et al.*, 1993*b*) suggest that the appropriate doses may be quite similar to those used in experiments on small animals. The tissue distribution of PPIX achieved with oral administration of ALA in experimental studies on the gastrointestinal tract was similar to that achieved with intravenous administration (Loh *et al.*, 1993*a*). Nevertheless, we feel that the use of ALA for PDT treatments of mucosal lesions of the larynx might be preferable with intravenous injection or infusion. The lower doses of ALA required (half that needed compared with oral administration, Loh *et al.*, 1993*a*) could be used more efficiently owing to the direct uptake in peripheral tissues avoiding the first-pass hepatic uptake probably encountered with oral administration. The choice of treatment time could also be easier. Endoscopic surgery on the larynx is normally performed under general anaesthesia and oral intake of fluids within 4 h of anaesthesia is not recommended. Nevertheless, oral administration of ALA is more convenient for patients undergoing endoscopy without general anaesthesia. Another factor is that the maximum dose of ALA that patients can tolerate by mouth is 60 mg $kg^{-1}$ (owing to hepatotoxicity after absorption from the gastrointestinal tract) and recent work suggests that this produces tissue levels of PPIX that are only just above the threshold required for a PDT effect (Messman *et al.*, 1995). The current experiments suggest that 100 mg $kg^{-1}$ intravenously is appropriate, but this would be equivalent to 200 mg $kg^{-1}$ orally, much more than can be tolerated by this route. A new preparation of ALA that can be given intravenously is required to assess what the maximum dose is that can be tolerated by this route before an appropriate dose for laryngeal tumours can be identified.

This study has identified conditions under which PDT can be used to produce mucosal necrosis in the normal larynx with safe healing by regeneration and no unacceptable changes in the submucosa, muscle or cartilage. Thus, PDT has potential for treating any lesion of the larynx in which the abnormal tissue has similar or greater susceptibility to PDT than the normal mucosa. Current evidence suggests that dysplasia and all tumours from carcinoma *in situ* to more invasive lesions of the upper aerodigestive tract are at least as susceptible as normal mucosa and so would be appropriate for PDT. Other possible targets would include preneoplastic lesions such as hyperplastic laryngitis, benign polyps, recurrences after radiotherapy and conditions such as laryngeal papillomatosis (Lofgren *et al.*, 1995). However, for PDT to eradicate lesions in the larynx, it is essential that the true extent of disease is known and that appropriate light doses can be delivered to all relevant areas, which may not always be straightforward. Nevertheless, PDT is minimally invasive and does appear to be safe, so if patients fail other conventional treatments such as radiotherapy or surgery it can still be given, which makes it an attractive first option, particularly in patients whose general condition is poor.

**Acknowledgements**

D Kleemann and T Mentzel were funded by the German Academic Exchange Service (DAAD) with additional support from DUSA Inc, Toronto. SG Bown is funded by The Imperial Cancer Research Fund. We should also like to thank J Bedwell and G Buonaccorsi for technical assistance.

### References

ABRAMSON AL, LEVY AS AND HIRSCHFIELD LS. (1990). The pathologic and thermal effects of gold vapor laser photodynamic therapy on the larynx. *Arch. Otolaryngol. Head Neck Surg.*, **116**, 687–691.

ABRAMSON AL, SHIKOWITZ MJ, MULLOOLY VM, STEINBERG BM, AMELLA CA AND ROTHSTEIN HR. (1992). Clinical effects of photodynamic therapy on recurrent laryngeal papillomas. *Arch. Otolaryngol. Head Neck Surg.*, **118**, 25–29.

BARR H, TRALAU CJ, BOULOS PB, MACROBERT AJ, KRASNER N, PHILLIPS D AND BOWN SG. (1990*a*). Selective necrosis in dimethylhydrazine-induced rat colon tumours using phthalocyanine photodynamic therapy. *Gastroenterology*, **98**, 1532–1537.

BARR H, MACROBERT AJ, TRALAU CJ, BOULOS PB AND BOWN SG. (1990*b*). The significance of the nature of the photosensitiser for photodynamic therapy:quantitative and biological studies in the colon. *Br. J. Cancer*, **62**, 730–735.

BEDWELL J, MACROBERT AJ, PHILLIPS D AND BOWN SG. (1992). Fluorescence distribution and photodynamic effects of ALA-induced PPIX in the DMH rat colonic tumour model. *Br. J. Cancer*, **65**, 818–824.

BERG K, BOMMER JC AND MOAN J. (1989). Evaluation of sulfonated aluminium phthalocyanines for use in photochemotherapy: cellular uptake studies. *Cancer Lett.*, **44**, 7–15.

BIEL MA. (1994). Photodynamic therapy and the treatment of neoplastic diseases of the larynx. *Laryngoscope*, **104**, 399–403.

BISHOP S, BEEBY A, KHOO BJ, MACROBERT AJ, SIMPSON MSC AND PHILLIPS D. (1993). Characterisation of the photochemotherapeutic agent disulphonated aluminium phthalocyanine using high performance liquid chromatography of separated components. *J. Chromatogr.*, **646**, 345–350.

BUGELSKI PJ, PORTER CW AND DOUGHERTY TJ. (1981). Autoradiographic distribution of HpD in normal and tumor tissue of the mouse. *Cancer Res.*, **41**, 4606.

CHAN WS, MACROBERT AJ, PHILLIPS D AND HART IR. (1989). Use of charged couple device for imaging of intracellular phthalocyanines. *Photochem. Photobiol.*, **50**, 617–624.

CHAN WS, MARSHALL JF, SVENSEN R, BEDWELL J AND HART IR. (1990). Effect of sulphonation on cell and tissue distribution of the photosensitiser aluminium phthalocyanine. *Cancer Res.*, **50**, 4533–4538.

CHATLANI PT, BEDWELL J, MACROBERT AJ, BARR H, BOULOS P, KRASNER N, PHILLIPS D AND BOWN SG. (1991). Comparison of di- and tetra- sulphonated aluminium phthalocyanines in normal rat colon. *Photochem. Photobiol.*, **53**, 745–751.

CHEVRETTON EB, BERENBAUM MC AND BONNET R. (1992). The effect of photodynamic therapy on normal skeletal muscle in an animal model. *Lasers Med. Sci.*, **7**, 103–110.

DE CORBIERE S, OUAYOUN M, SEQUERT C, FRECHE CH AND CHABOLLE F. (1992). Use of photodynamic therapy in the treatment of vocal cord carcinoma. Retrospective study 1986–1992 on 41 cases. In *Photodynamic Therapy and Biomedical Lasers*, Spinelli P, Dal Fante M, Marchesini R. (eds) pp. 656–661. Elsevier Science: Amsterdam.

DIVARIS DSG, KENNEDY JC AND POTTIER RH. (1990). Phototoxic damage to sebaceous glands and hair follicles of mice after systemic administration of 5-aminolevulinic acid correlates with localized protoporphyrin IX fluorescence. *Am. J. Pathol.*, **136**, 891–897.

FEYH J. (1992). The treatment of larynx papillomas with the aid of Photodynamic therapy. In *Photodynamic Therapy and Biomedical Lasers*, Spinelli P, Dal Fante M, Marchesini R. (eds) pp. 653-655. Elsevier Science: Amsterdam.

FEYH J, GUTMANN R AND LEUNIG A. (1993). Die photodynamische Lasertherapie im Bereich der Hals-, Nasen-, Ohren heilkunde. *Laryngo-Rhino-Otol*, **72,** 273-278.

FEYH J, GOETZ A, MÜLLER W, KÖNIGSBERGER R AND KASTENBAUER E. (1990). Photodynamic therapy in head and neck surgery. *J. Photochem. Photobiol.*, **7,** 353-358.

FRECHE CH AND DE CORBIERE S. (1990). Use of photodynamic therapy in the treatment of vocal cord carcinoma. *J. Photochem. Photobiol.*, **6,** 291-296.

GLUCKMANN JL AND WEISSLER MC. (1986). Role of photodynamic therapy in the management of early cancers of the upper aerodigestive tract. *Lasers Med. Sci.*, **1,** 217-220.

GRANT WE, HOPPER C, SPEIGHT P, MACROBERT AJ AND BOWN SG. (1993*a*). Photodynamic therapy of malignant and premalignant lesions in patients with 'field cancerization' of the oral cavity. *J. Laryngol. Otol.*, **107,** 1140-1145.

GRANT WE, HOPPER C, MACROBERT AJ, SPEIGHT PM AND BOWN SG. (1993*b*). Photodynamic therapy of oral cancer: photosensitisation with systemic aminolaevulinic acid. *Lancet*, **342,** 147-148.

JUDD MD, BEDWELL J, MACROBERT AJ AND BOWN SG. (1992). Comparison of the distribution of phthalocyanine and ALA-induced porphyrin sensitisers within the rabbit uterus. In *Photodynamic Therapy and Biomedical Lasers*, Spinelli P, Dal Fante M, Marchesini R. (eds) pp. 322-326. Elsevier Science: Amsterdam.

KENNEDY JC AND POTTIER RH. (1992). Endogenous protoporphyrin IX, a clinically useful photosensitizer for photodynamic therapy. *J. Photochem. Photobiol. B: Biol*, **14,** 275-292.

KENNEDY JC, POTTIER RH AND PROSS DC. (1990). Photodynamic therapy with endogenous protoporphyrin IX: basic principles and present clinical experience. *J. Photochem. Photobiol. B: Biol.*, **6,** 143-148.

KLEEMANN D. (1990). Experimentelle Untersuchungen zur Photodynamischen Therapie von malignen Tumoren der Mundhöhle, des Larynx und Pharynx mit dem Photosensibilisator Methylenblau. *Laryngo-Rhino-Otol.*, **69,** 437-439.

KLEINSASSER O. (1987). *Tumoren des Larynx und des Hypopharynx*. pp. 159-164. George Thieme: Stuttgart.

LOFGREN LA, RONN AM, NOURI M, LEE CJ, YOO D AND STEINBERG BM. (1995). Efficacy of intravenous 5-amino laevulinic acid photodynamic therapy on rabbit papillomas. *Br. J. Cancer*, **72,** 857-864.

LOH CS, BEDWELL J, MACROBERT AJ, KRASNER N, PHILLIPS D AND BOWN SG. (1992). Photodynamic therapy of the normal rat stomach: a comparative study between di-sulphonated aluminium phthalocyanine and 5-aminolaevulinic acid. *Br. J. Cancer*, **66,** 452-462.

LOH CS, MACROBERT AJ, BEDWELL J, REGULA J, KRASNER N AND BOWN SG. (1993*a*). Oral versus intravenous administration of 5-aminolaevulinic acid for photodynamic therapy. *Br. J. Cancer*, **68,** 41-51.

LOH CS, VERNON D, MACROBERT AJ, BEDWELL, BOWN, SG AND BROWN SB. (1993*b*). Endogenous porphyrin distribution induced by 5-aminolaevulinic acid in the tissue layers of the gastrointestinal tract. *J. Photochem. Photobiol. B: Biol.*, **20,** 47-54.

MALIK Z AND LUGACI H. (1987). Destruction of erythroleukaemic cells by photoactivation of endogenous porphyrins. *Br. J. Cancer*, **56,** 589-595.

MARRIOTT J. (1968) Regulation of porphyrin synthesis. *Biochem. Soc. Symp.*, **28,** 61-74.

MESSMAN H, MLKVY P, BUONACCORSI G, DAVIES CL, MACROBERT AJ AND BOWN SG. (1995). Enhancement of photodynamic therapy with 5-aminolaevulinic acid-induced porphyrin photosensitisation in normal rat colon by threshold and light fractionation studies. *Br. J. Cancer*, **72,** 589-594.

MEYER M, SPEIGHT P AND BOWN SG. (1991). A study of the effects of photodynamic therapy on the normal tissue of the rabbit jaw. *Br. J. Cancer*, **64,** 1093-1097.

MONNIER PH, SAVARY M, FONTOLLIET CH, WAGNIERES G, CHATELAIN A, CORNAZ P, DEPEURSINGE CH AND VAN DEN BERGH H. (1990). Photodetection and photodynamic therapy of 'early' squamous cell carcinomas of the pharynx, oesophagus and tracheo-bronchial tree. *Lasers Med. Sci.*, **5,** 149-168.

NUUTINEN PJO, CHATLANI PT, BEDWELL J, MACROBERT AJ, PHILLIPS D AND BOWN SG. (1991). Distribution and photodynamic effect of disulphonated aluminium phthalocyanine in the pancreas and adjacent tissues in the Syrian golden hamster. *Br. J. Cancer*, **64,** 1108-1115.

PAQUETTE B, ALI H, LANGLOIS R AND VAN LIER VE. (1988). Biological activities of phthalocyanines VIII. Cellular distribution in V-79 Chinese hamster cells and phototoxicity of selectively sulfonated aluminium phthalocyanines. *Photochem. Photobiol.*, **47,** 215-220.

PENG Q, MOAN J, WARERLOE T, NESLAND JM AND RIMINGTON C. (1992). Distribution and photosensitizing efficiancy of porphyrins induced by application of exogenous 5-aminolaevulinic acid in mice bearing mammary carcinoma. *Int. J. Cancer*, **52,** 433-443.

POPE AJ AND BOWN SG. (1991). The morphological and functional changes in rat bladder following photodynamic therapy with phthalocyanine photosensitization. *J. Urol.*, **145,** 1064-1070.

REGULA J, MACROBERT AJ, GORCHEIN A, BUONACCORSI GA, THORPE SM, SPENCER GM, HATFIELD ARW AND BOWN SG. (1995). Photosensitisation and photodynamic therapy of oesophageal, duodenal and colorectal tumours using 5-aminolaevulinic acid induced protoporphyrin IX: a pilot study. *Gut*, **36,** 67-75.

RIMINGTON C. (1966). Porphyrin and haem biosynthesis and its control. *Acta Med. Scand.*, **179,** 11-24.

SMITH SGT, BEDWELL J, MACROBERT AJ, GRIFFITHS MH, BOWN SG AND HETZEL MR. (1993). Experimental studies to assess the potential of photodynamic therapy for the treatment of bronchial carcinomas. *Thorax*, **48,** 474-480.

TRALAU CJ, BARR H, SANDEMAN DR, BARTON T, LEWIN MR AND BOWN SG. (1987). Aluminium sulphonated phthalocyanine distribution in rodent tumours of the colon, brain and pancreas. *Photochem. Photobiol.*, **46,** 777-781.

TRALAU CJ, YOUNG AR, WALKER NPJ, VERNON DI, MACROBERT AJ, BROWN SB AND BOWN SG. (1989). Mouse skin photosensitivity with dihaematoporphyrin ether (DHE) and aluminium sulphonated phthalocyanine (AlSPc): a comparative study. *Photochem. Photobiol.*, **49,** 305-312.

WOLF P AND KERL H. (1991). Photodynamic therapy in patients with xeroderma pigmentosum. *Lancet*, **337,** 1613-1614.

WUSTROW TPU, JOCHAM D, SCHRAMM A AND UNSÖLD E. (1988). Photodynamische Zerstörung *in vitro* kultivierter Plattenepithelkarzinomzellen aus dem Kopf-Hals-Bereich. *Laryngo-Rhino- Otol.*, **67,** 532-538.

WUSTROW TPU, SCHRAMM A, JOCHAM D AND UNSÖLD E. (1989). Laserlicht-verursachte Zytotoxizität kultivierter Plattenepithelkarzinomzellen aus dem Kopf-Hals-Bereich nach Photosensibilisierung. *Laryngo-Rhino-Otol.* **68,** 44-50.